# 3D bioprinted tumor model: a prompt and convenient platform for overcoming immunotherapy resistance by recapitulating the tumor microenvironment

**DOI:** 10.1007/s13402-024-00935-9

**Published:** 2024-03-23

**Authors:** Zhanyi Zhang, Xuebo Chen, Sujie Gao, Xuedong Fang, Shengnan Ren

**Affiliations:** 1 Bethune Third Clinical Medical College, Jilin University, Changchun, 130021 China; 2 Department of Gastrointestinal, Colorectal and Anal Surgery, China-Japan Union Hospital of Jilin University, NO. 126, Xiantai Street, Changchun, 130033 China; 3 Department of Anesthesiology, China-Japan Union Hospital of Jilin University, Changchun, 130033 China; 4 Department of Breast Surgery, Peking University Cancer Hospital Yunnan, Yunnan Cancer Hospital, The Third Affiliated Hospital of Kunming Medical University, NO. 519, Kunzhou Street, Kunming, 650118 China

**Keywords:** Bioprinting, In vitro tumor model, Cancer immunotherapy, Acquired resistance, Personalized medication

## Abstract

**Background:**

Cancer immunotherapy is receiving worldwide attention for its induction of an anti-tumor response. However, it has had limited efficacy in some patients who acquired resistance. The dynamic and sophisticated complexity of the tumor microenvironment (TME) is the leading contributor to this clinical dilemma. Through recapitulating the physiological features of the TME, 3D bioprinting is a promising research tool for cancer immunotherapy, which preserves in vivo malignant aggressiveness, heterogeneity, and the cell–cell/matrix interactions. It has been reported that application of 3D bioprinting holds potential to address the challenges of immunotherapy resistance and facilitate personalized medication.

**Conclusions and Perspectives:**

In this review, we briefly summarize the contributions of cellular and noncellular components of the TME in the development of immunotherapy resistance, and introduce recent advances in 3D bioprinted tumor models that served as platforms to study the interactions between tumor cells and the TME. By constructing multicellular 3D bioprinted tumor models, cellular and noncellular crosstalk is reproduced between tumor cells, immune cells, fibroblasts, adipocytes, and the extracellular matrix (ECM) within the TME. In the future, by quickly preparing 3D bioprinted tumor models with patient-derived components, information on tumor immunotherapy resistance can be obtained timely for clinical reference. The combined application with tumoroid or other 3D culture technologies will also help to better simulate the complexity and dynamics of tumor microenvironment in vitro. We aim to provide new perspectives for overcoming cancer immunotherapy resistance and inspire multidisciplinary research to improve the clinical application of 3D bioprinting technology.

## Introduction

Since William Coley first leveraged the immune system to eradicate cancer using Streptococcus pyogenes and Serratia marcescens in 1891, immunotherapy has received increasing attention and flourished with the development of immune checkpoint inhibitors (ICIs) and adoptive cell transfer (ACT) T-cell therapies [1]. So far, evidence has justified the clinical benefit of ICI, ACT, and other immunotherapies in provoking an anti-tumor immune response to control cancer progression and metastasis [2, 3]. However, efficacy generally exists in a subset of cancer patients, but a durable response is difficult to sustain as acquired resistance may develop. The underlying mechanism of acquired resistance can be categorized into two aspects: constantly evolving tumor expression profile and immunosuppressive network forming between cancerous cells and non-cancerous cells in the microenvironment.

Tumor cells constantly evolve to change their expression profile to lose neoantigens, reduce mutation burden, and secrete suppressive molecules, facilitating immune evasion and promoting malignant growth/metastasis [4, 5]. The tumor microenvironment (TME) is critical in establishing an immunosuppressive background with the cellular and non-cellular components. A complex and dynamic network is constructed by tumor-infiltrating lymphocytes (TILs), including cytotoxic CD8+ T cells, CD4+ Teffs, T regulatory cells (Tregs), B cells, tumor-associated macrophages (TAMs), myeloid-derived suppressor cells (MDSCs), and non-immune cells, including cancer-associated fibroblasts (CAFs) and tumor endothelial cells (TECs). The communications and interactions between tumor, immune, and non-immune cells contribute to tumor heterogeneity and the generation of resistance to immunotherapies [6]. Moreover, the stiffness of the extracellular matrix (ECM) structure and increased tumor interstitial fluid pressure (IFP) are responsible for impaired treatment effectiveness [7]. In this case, two-dimensional (2D) cell culture models are inadequate for elucidating the underlying mechanisms of immunotherapy resistance owing to the loss of crucial phenotypes of tumor cells and altered responses to immunotherapies [8]. In contrast, three-dimensional (3D) models better mimic the complexity of the TME and crosstalk between tumor cells and the TME [9].

Three-dimensional 3D in vitro models include spheroids, biopolymer scaffolds, ex vivo tissue slices, organs-on-chips, and 3D bioprinting. Among these, 3D bioprinting is a reproducible technology with several advantages: it can be precisely controlled, and the spatial deposition of multiple types of cells can be accurately defined in a prompt, convenient, and high-throughput manner. Bioinks refer to cell-laden solutions containing various types of living cells suspended in biocompatible materials, which can be printed using several methods [10]. Natural materials (e.g. alginate, gelati, and hyaluronic acid) and synthetic biomaterials (e.g. PCL, PEG, and Pluronic) are commonly used to prepare bioinks, as well as extracellular matrix (ECM) is also recommended to reproduce biomimetic tumor models [11, 12]. 3D bioprinted in vitro models have achieved remarkable progress in tissue engineering and drug screening [13, 14]. As for 3D bioprinted tumor models, tumor cells exhibit different expression profiles associated with enhanced aggressive behavior compared with those in 2D culture models but share similarities to those cultured in xenograft models [15]. Moreover, researchers have successfully constructed biomimetic bioprinted model with patient-derived tumor cells and ECM, making it possible to realize personalized treatment [16, 17]. In terms of mimicking the TME, 3D bioprinting has been used to create an organized milieu for investigating the interplays between different cell types and a replicated model for tumor metastasis and angiogenesis [18]. In this review, we briefly introduce the immunosuppressive network formed in the TME, which contributes to developing acquired resistance to immunotherapy. Subsequently, advances in 3D bioprinted tumor models are summarized, which are constructed to study the interactions between tumors and the TME. These models represent a prompt and convenient platform to investigate cancer immunotherapy resistance. We aim to highlight 3D bioprinting as a promising technology to study the complex and dynamic network between cellular and noncellular components in the TME. This will improve our understanding of cancer immunotherapy resistance and provide a valuable tool to realize personalized medicine.

## Immunosuppressive network in the TME

### Tumor cell-mediated crosstalk

Tumor cells are involved in continuous, chronic, and dynamic evolution from initiation to metastasis. By altering the molecular expression profile, tumor cells are the leading cause of acquired immunotherapy resistance. On the one hand, epigenetic and genetic alterations impair neoantigen transcription and translation, causing the loss of neoantigen expression. These neoantigens include selected targets for cancer vaccines and ACT. With CD19-CAR T-cell therapy, acquired resistance developed in a diffuse large B-cell lymphoma, which was associated with the loss of CD19 expression [19]. In non-small cell lung cancer (NSCLC), the loss of neoantigens associated with T-cell activation was observed in tumors that developed resistance to ICIs [20]. On the other hand, tumor cells express suppressive molecules (such as indoleamine 2,3-dioxygenase (IDO), adenosine, and CCL2) to inhibit T-cell activity and recruit Tregs to tumor sites, impairing immunotherapy efficacy [21–23]. Tumor-derived exosomes (TEXs), which contain many immunosuppressive molecules, also contribute to crosstalk between tumor and immune cells within the TME [24].

### Immune cell-mediated crosstalk

Dysfunction of cytotoxic T cells and recruitment of suppressive immune cells are the major reasons for acquired resistance to immunotherapy. By expressing and secreting many soluble molecules, immunosuppressive cells communicate with each other and generate a suppressive network within the TME, which weakens the power of immunotherapy and facilitates tumor immune evasion. First, the upregulation of ICs in immune cells is observed after blocking one IC due to the compensatory inhibitory mechanism. Anti-PD-1/PD-L1 treatment typically leads to increased TIM-3+ T-cell infiltration and upregulation of LAG-3 in CD8+ T cells [25–27]. Second, suppressive soluble molecules released by immune cells impair the anticancer immune response and facilitate tumor growth/metastasis. Tregs, TAM, and MDSCs, are predominant sources of these factors, including chemokines, growth factors, cytokines, and proteases [28, 29]. Moreover, some dendritic cell (DC) subsets increase IDO expression, with negative modulation of the anticancer immune response [21]. Finally, crosstalk between suppressive and other immune cells is responsible for acquired resistance to immunotherapy. Through secretion of IL-9, IL-10, and adenosine, Tregs directly or indirectly interact with TAM, MDSC, and TAMCs, which inhibit cytotoxicity and effector T-cell function and further increase the number of immunosuppressive cells in the TME [30–33]. Interactions between B regulatory cells (Bregs), MDSCs, and Tregs have also been reported in breast cancer and squamous cell carcinoma, which increased IL-10 production and PD-1 expression in Bregs, enhancing the expansion of Tregs in the TME [34, 35]. Crosstalk between TAM and tumor cells has been confirmed by the positive feedback loop that induces the epithelial–mesenchymal transition (EMT) of tumor cells and secretes GM-CSF and CCL18 [36]. Upon recruitment to the TME by colorectal cancer cells, TAMCs facilitate colorectal cancer growth and metastasis via TLR-2-mediated activation [37].

### Non-immune stromal cell-mediated crosstalk

CAFs are one of the most important stromal cells in the TME, and they participate in developing immunotherapy resistance through a direct and indirect interplay with immunosuppressive and tumor cells. By secreting IL-8, CAFs promote the differentiation of monocytes into M2-type TAMs and suppress NK cell activity in the TME [38]. By interacting with tumor cells, CAFs release IL-6 and TGF-β to increase the number and activity of Tregs in the TME. Meanwhile, a positive feedback loop forms in which TGF-β stimulates the differentiation of normal resident fibroblasts into CAFs, enhancing the immunosuppressive network within the TME [39–41]. Tumor endothelial cells (TECs) also interact with tumor cells by releasing VEGF, which promotes angiogenesis, facilitating tumor immune evasion [6].

### Biophysical factors

**Fig. 1:**
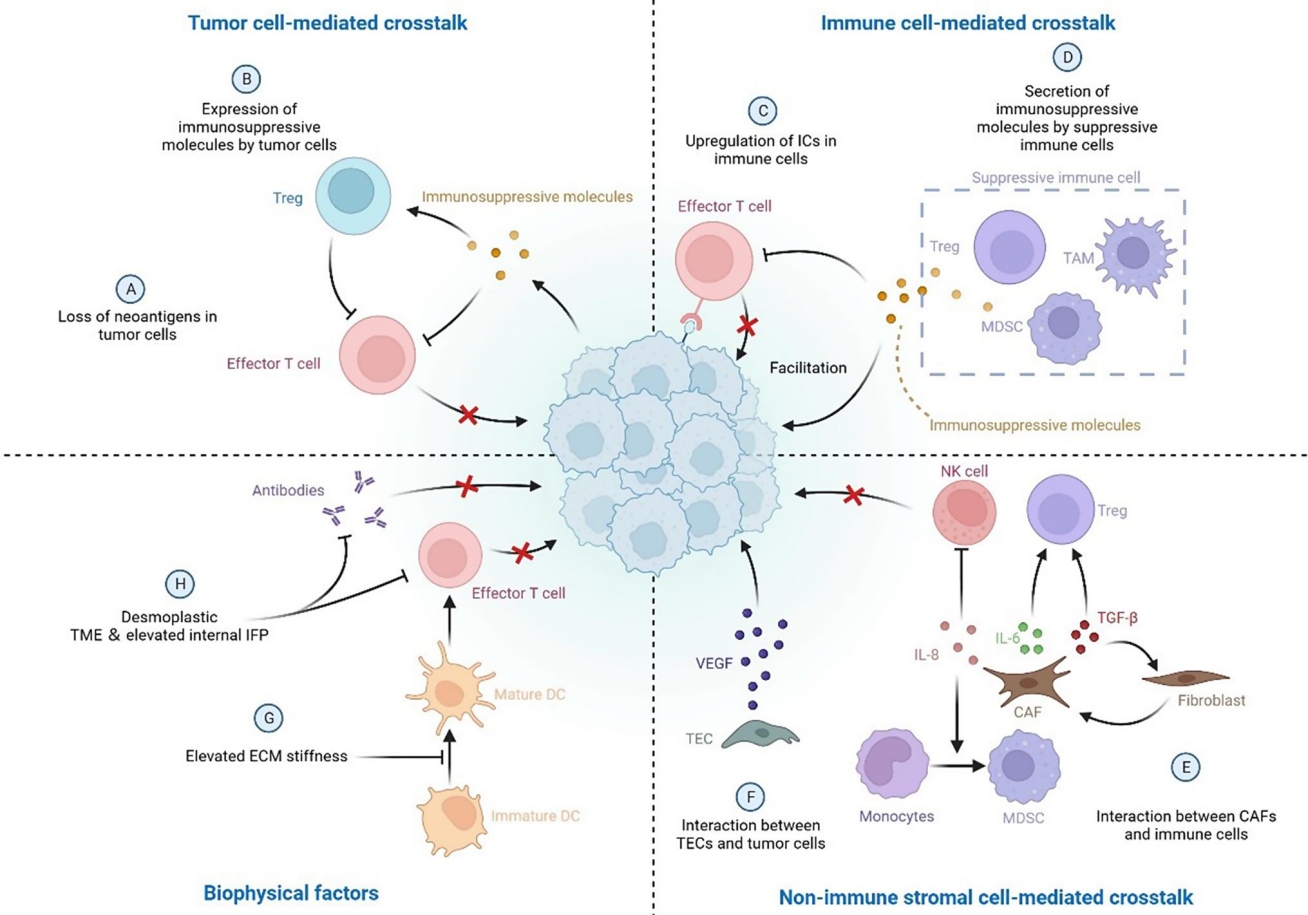
Schematic illustration of immunosuppressive network in the TME. *Treg* T regulatory cell. *ICs* immune checkpoints. *TAM* tumor-associated macrophage. *MDSC* myeloid-derived suppressor cells. *NK cell* natural killer cell. *TGF-β* Transforming growth factor beta. *IL-6/8* interleukin 6/8. *CAF* cancer-associated fibroblast. *VEGF* vascular endothelial-derived growth factor. *TEC* tumor endothelial cell. *DC* dendritic cell. *ECM* extracellular matrix. *IFP* interstitial fluid pressure. *TME* tumor microenvironment. Created with Biorender.com

Recent advances in functional biomaterials and micro/nanotechnologies have revealed that the biophysical cues of the TME (including ECM structure, ECM stiffness, IFP, solid stress, and vascular shear) play crucial roles in determining tumor immune properties [42–44]. These biophysical factors facilitate the development of immunotherapy resistance primarily by affecting the biological behaviors of immune cells and interfering with the integrity of the anti-tumor immunity cascade. The elevated ECM stiffness within the TME inhibits the podosome formation and antigen recognition of DC [45]. Meanwhile, CD8^+^ T-cell trafficking into the tumor site may be blocked by the prominent desmoplastic TME, which also causes vascular dysfunction to hinder therapeutic antibody delivery, resulting in an immunosuppressive microenvironment [46–48]. In some solid tumors, intertumoral stress increases considerably as malignant cells proliferate rapidly, which induces the expression of mechanical sensing proteins activating the pro-tumoral signaling pathway in tumor-repopulating cells [49]. Elevated internal IFP can generate a huge pressure gradient in the tumor margin and dramatically affect cell behaviors induced by mechanical shearing [50]. Notably, solid stress elevates the IFP, which enhances the solid stress of the tumor reciprocally by flow-induced stiffening [42]. Moreover, the desmoplastic reaction can generate exceedingly high IFP and cause substantial inhibition of drug delivery [51]. The schematic illustration of immunosuppressive network in the TME is demonstrated in Fig. 1.

## 3D bioprinting

### Bioprinting strategies

3D bioprinting (additive manufacturing) is emerging as an ideal tool for building complicated 3D cancer models. It enables 3D biological structures by depositing cell-laden biomaterials based on predefined programs layer-by-layer via a bottom-up assembly approach [52]. Commonly used techniques for 3D bioprinting include extrusion-based, droplet-based, laser-assisted, and aspiration-based bioprinting. The principles and characteristics of each bioprinting technique were summarized by Murphy et al [53]. Generally, extrusion-based bioprinting (EBB) uses air pressure, screws, or mechanical pistons to extrude bioinks from a nozzle into desired patterns [52]. This strategy preserves cell viability up to as high as 81.5% after 24 h of incubation and is the most commonly used method for biological applications [54]. The shortcomings of EBB include low resolution and slow printing speed for large structures [55]. Droplet-based bioprinting (DBB), also known as inkjet-based bioprinting, is a technology that deposits cell-containing bioink droplets precisely onto a supporting material. This strategy allows the construction of structures with high resolution at a low cost. DBB can be effectively used to print highly complex combinations of biomaterials (*e.g.*, hydroxyapatite, polyethyleneimine, and riboflavin sodium phosphate) to create more informative cancer models, providing an ideal candidate for bio-imaging and drug delivery [56]. Nonetheless, this printing strategy requires low-viscosity bioinks to achieve ideal droplet deposition, which may negatively influence print fidelity and cell encapsulation efficiency [57, 58]. Laser-assisted bioprinting (LAB) utilizes lasers focused on a photosensitive substrate to generate pressures, and the two widely used methods are laser-induced forward transfer and digital light processing (DLP). LAB is nozzle-free to avoid cell-clogging issues with exceptional precision and high throughput. However, LAB is complex and can only utilize limited materials. The bioprinting process with laser energy is slower than other methods, and the laser may impair cell viability, leading to low cell survival rates [52]. Aspiration-based bioprinting (ABB) has been recommended as an effective strategy to construct 3D in vitro models with aspiration and bioprinting physical forces to pick up and deposit tiny tissue blocks, such as spheroids [59]. Thus, ABB is considered an effective strategy involving simple procedures, relatively low cost, and reproducibility, which can be used in various applications.

Recently, Zhou et al. proposed that 3D bioprinting can also be categorized into basic cell bioprinting and advanced aggregate bioprinting depending on the assembly method of bioink [9]. As the name suggests, basic cell bioprinting is generally processed using cell suspensions, loaded into hydrogel biomaterials, and printed into 3D constructs on demand. The hydrogel biomaterials provide mechanical support and serve as the matrix. The cells are confined, grow, and proliferate in a lattice array formed by a crosslinking network from hydrogel biomaterials. In contrast, aggregate bioprinting has been recently utilized to establish more complex tumor models, where cells are first cultured in aggregates, including organoids and spheroids, and then assembled to form an advanced tumor model. Cells are no longer confined in the polymeric lattice but form direct connections with surrounding cells and produce the ECM during metabolism. Aggregate bioprinting involves other stromal components in building a more sophisticated 3D model with a higher cell density and a multicellular structure. It is more complex than organoids and better recapitulates the pathological features and cellular interactions of tumors in vivo [60]. With a high throughput and perfect simulation of the dynamic tumor microenvironment, 3D bioprinted tumor models have been widely used as a promising strategy to study the cellular and noncellular crosstalk within the TME and to screen sensitive drugs and treatments. For applications of 3D bioprinting for cancer drug screening, we refer readers to these excellent reviews [14, 61–63]. In the following section, we will thoroughly discuss the superiority of the 3D bioprinted tumor model in recapitulating tumor aggressiveness and simulating crosstalk between tumor cells and immune cells, nonimmune cells, and the surrounding matrix (Table 1).

**Table 1:** Summary of 3D bioprinted tumor models

Bioprinting technology	Bioink	Cellular components	References
Extrusion-based bioprinting	Cell-laden gelatin/alginate hydrogel	Breast cancer stem cells	[62]
	Sodium alginate/gelatin hydrogel	Glioma stem cell GSC-23	[63]
	Gelatin-alginate-fibrinogen hydrogel	Glioma cells U118	[64]
	Hydrogel	Glioma stem cell GSC-23 and U118	[65]
	Gelatin and sodium alginate	Patient-derived HCC cells	[13]
	Alginate	Breast cancer cell (ZR75.1)	[11]
	Alginate	Breast cancer cells MDA-MB-231 and macrophages	[66]
	Gelatin methacryloyl (GelMA) and gelatin	Mouse glioblastoma cells GL261 and mouse macrophage cell line RAW264.7	[67]
	Gelatin/alginate hydrogel	mM1 murine pancreatic ductal adenocarcinoma cells and pancreatic cancer associated fibroblasts	[68]
	HA-based hydrogels	MDA-MB-231 cells and adipose-derived stem cells	[69]
	GelMA	Cholangiocarcinoma cells and stromal cells	[70]
	Matrigel	Patient-derived tumor cells, normal bladder stem cells, CAFs, epithelial cells, immune cells, and smooth muscle cells	[56]
	Fibrinogen and gelatin	Patient-derived glioblastoma cells, astrocytes, microglia, brain pericytes, and endothelial cells	[71]
	Mammary-derived ECM hydrogel	Breast cancer cell MCF-7 and MDA-MB-231	[72]
Droplet-based bioprinting	Hydrogel mixed with CELLINK Bioink, CELLINK RGD10, CELLINK Laminink111, CELLINK Laminink411 or CELLINK Laminink521 hydrogels	CLL cells and MEC1 cells	[12]
Laser-assisted bioprinting	GelMA	CAR T cells and neuroblastoma cells	[73]
	Alginate/gelatin hydrogel	Breast cancer cells and differentiated adipocytes	[74]
	GelMA and nHA	Breast cancer cells and MSCs	[75]
	GelMA, polyethylene glycol diacrylate (PEGDA) ink, and nHA	Breast cancer cells, osteoblasts, and human umbilical vein endothelial cells	[76]
	GelMA hydrogel	Acinar and ductal cells	[77]
	HA-rich hydrogel	Patient-derived GSCs, macrophages, and nonimmune cells (astrocytes and neural stem cells)	[78]
	GMHA and GelMA	Patient-derived GBM cells and human endothelial cells	[79]

### Recapitulating in vivo tumor aggressiveness and heterogeneity

Generally, tumor cells exhibit more invasive phenotypes when cultured in 3D models than in 2D plates or Petri dishes [64]. 3D bioprinting facilitates the stemness maintenance and heterogeneity of patient-derived tumor cells. By extrusion printing, Hong et al. constructed a cross-shaped architecture with a cell-laden gelatin/alginate hydrogel [65]. They observed that the breast cancer stem cells sustained the drug-resistant phenotype of CD44^high^/CD24^low^/ALDH1^high^ in a 3D bioprinted model. Meanwhile, the sensitivity to anticancer agents was also reduced. Wang et al. recently established an in vitro 3D model with glioma stem cells (GSC23) suspended in a sodium alginate/gelatin hydrogel by extrusion printing [66]. Compared with a traditional cell suspension culture, 3D bioprinted GSC-23 exhibited a more stable proliferation status, and the viability was 86.27 ± 2.41% after bioprinting. Additionally, microvilli formation and increased VEGFA secretion were observed in the 3D bioprinting model, indicating a stronger angiogenesis potential. In another study, the same researchers constructed a 3D in vitro model with glioma U118 cells and a gelatin–alginate–fibrinogen hydrogel using the same printing method [67]. Compared with cells cultured under 2D conditions, the stemness properties were increased in 3D bioprinted models, including a higher proportion of CD133^+^ glioma cells, upregulated EMT, and increased in vivo tumorigenicity. Furthermore, the same team investigated coaxial extrusion bioprinting to construct hydrogel microfibers with glioma stem cells (GSC23) as the shell and U118 cells as the core [78]. In this model, fiber-like aggregates were produced, and cell–cell and cell–ECM interactions formed. U118 cells exhibited a more aggressive phenotype and higher drug resistance-related gene expression.

**Fig. 2:**
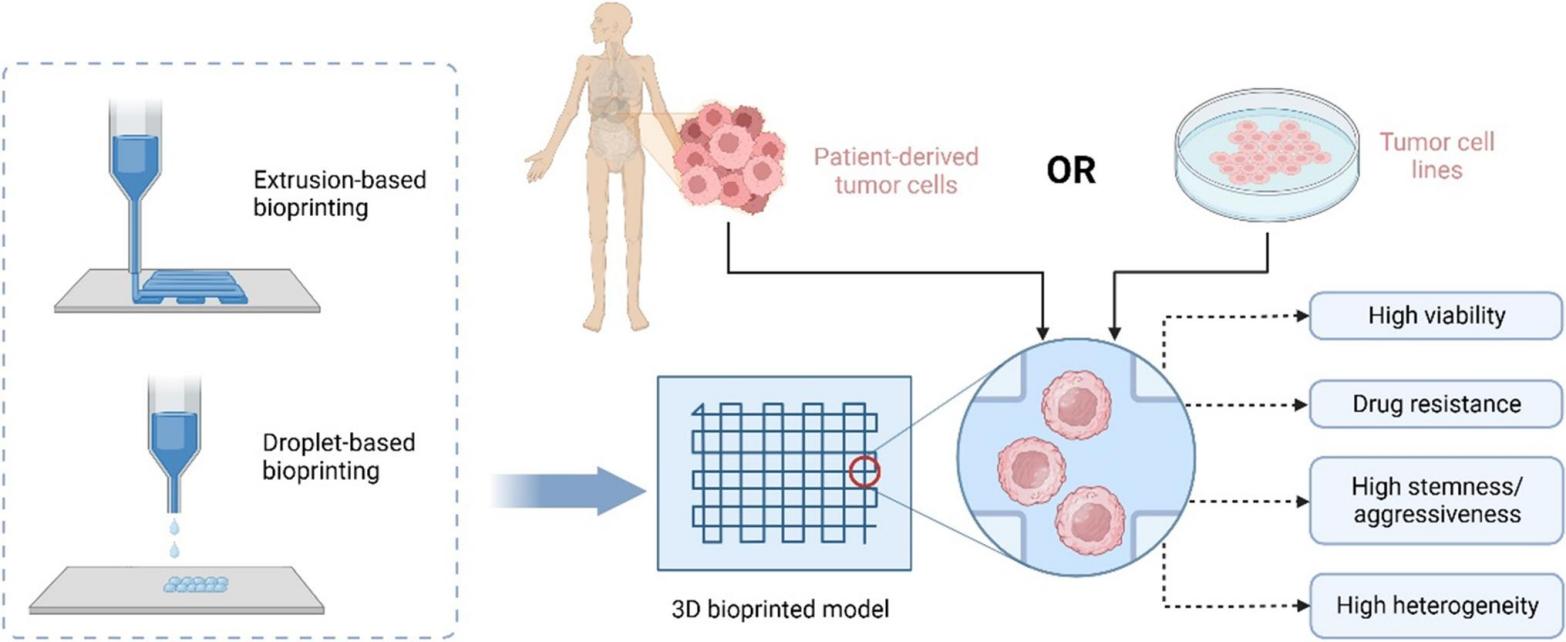
3D bioprinted tumor model recapitulates in vivo tumor aggressiveness and heterogeneity. Created with Biorender.com

Alternatively, patient-derived cells can be bioprinted to recapitulate the unique pathological features of an individual tumor. Sbrana et al. suspended patient-derived chronic lymphocytic leukemia (CLL) and MEC1 cells in a hydrogel to establish a long-term 3D culture CLL model [16]. The patient-derived CLL cells sustained expression of CD19 and CD5 and increased levels of IgM. Recently, Xie et al. established a hepatocellular carcinoma (HCC) 3D bioprinted model with patient-derived HCC cells isolated from six hepatectomy specimens [17]. Primary HCC cells were used to construct patient-derived 3D bioprinted HCC (3DP-HCC) models by mixing with gelatin and sodium alginate. During long-term culture, these patient-derived HCC cells preserved the genetic and phenotypic characteristics of the original tumors. Moreover, the sensitivity to the tested drugs was consistent with mutant targets detected by whole exon sequencing (WES). Recently, Dankó et al. reported a comparative analysis to study the metabolic heterogeneity in 3D bioprinted and xenograft breast cancer models [15]. Luminal breast cancer cell (ZR75.1)-laden alginate-based bioink was utilized to construct a 3D culture model by extrusion bioprinting. By immunohistochemistry staining, the expression patterns and heterogeneous staining of metabolic proteins (p-mTOR, FASN, p-ACC, and p-S6) showed remarkable similarity in a 3D bioprinted tissue mimetic system and xenograft models. Preservation of tumor aggressiveness and heterogeneity by 3D tumor model is demonstrated in Fig. 2.

### Simulation of crosstalk between tumor cells and immune cells

In 3D bioprinted tumor models, the interactions between immune and tumor cells show that immune cells are recruited by tumor cells, infiltrated/dispersed into tumor sites, and activated/differentiated by tumor cells. Grolman et al. designed a co-culture alginate fiber containing MDA-MB-231 breast cancer cells in alginate and macrophages in CaCl_2_ at the center by extrusion bioprinting [73]. Owing to the cellular interaction mediated by the epidermal growth factor receptor (EGFR) on cancer cells, macrophages were recruited to intersperse among the entire hydrogel after culture for 4 days. Recently, Heinrich et al. reported an exceptional 3D bioprinting strategy to illustrate the crosstalk between tumor cells and macrophages [68]. The 3D bioprinted mini-brains were fabricated by GL261 mouse glioblastoma cells in the cavity surrounded by the RAW264.7 mouse macrophage cell line. They were all suspended in bioink consisting of gelatin methacryloyl (GelMA) and gelatin. This delicate 3D bioprinted mini-brain demonstrated that paracrine and juxtacrine signaling was responsible for the interaction between tumor cells and macrophages, which drove the migration and polarization of macrophages into the tumor site. Moreover, the expression profiles of GL261 and RAW264.7 underwent considerable changes compared with those cultured in the 2D monolayer, including upregulated Spp1, loss of E-cadherin, and increased Fgf2. A more sophisticated 3D bioprinting model was reported by Tang et al., who employed a DLP-based rapid 3D bioprinting system and photo-cross-linkable native ECM derivatives to construct a multicellular biomimetic environment for glioblastoma [74]. It included patient-derived GSCs, macrophages, and nonimmune cells (astrocytes and neural stem cells) suspended in an HA-rich hydrogel. According to RNA sequencing and bioinformatics analysis, the 3D tetra-culture system (GSC, macrophages, astrocytes, and neural stem cells) recapitulated well the expression profile of glioblastoma tissues. The system exhibited upregulated cellular interaction, hypoxia, and cancer stem cells in contrast to a 3D tri-culture system without macrophages. Importantly, this 3D bioprinted multicellular tumor model signified the importance of macrophages in activating the extracellular matrix and sustaining the aggressiveness of tumor cells.

**Fig. 3:**
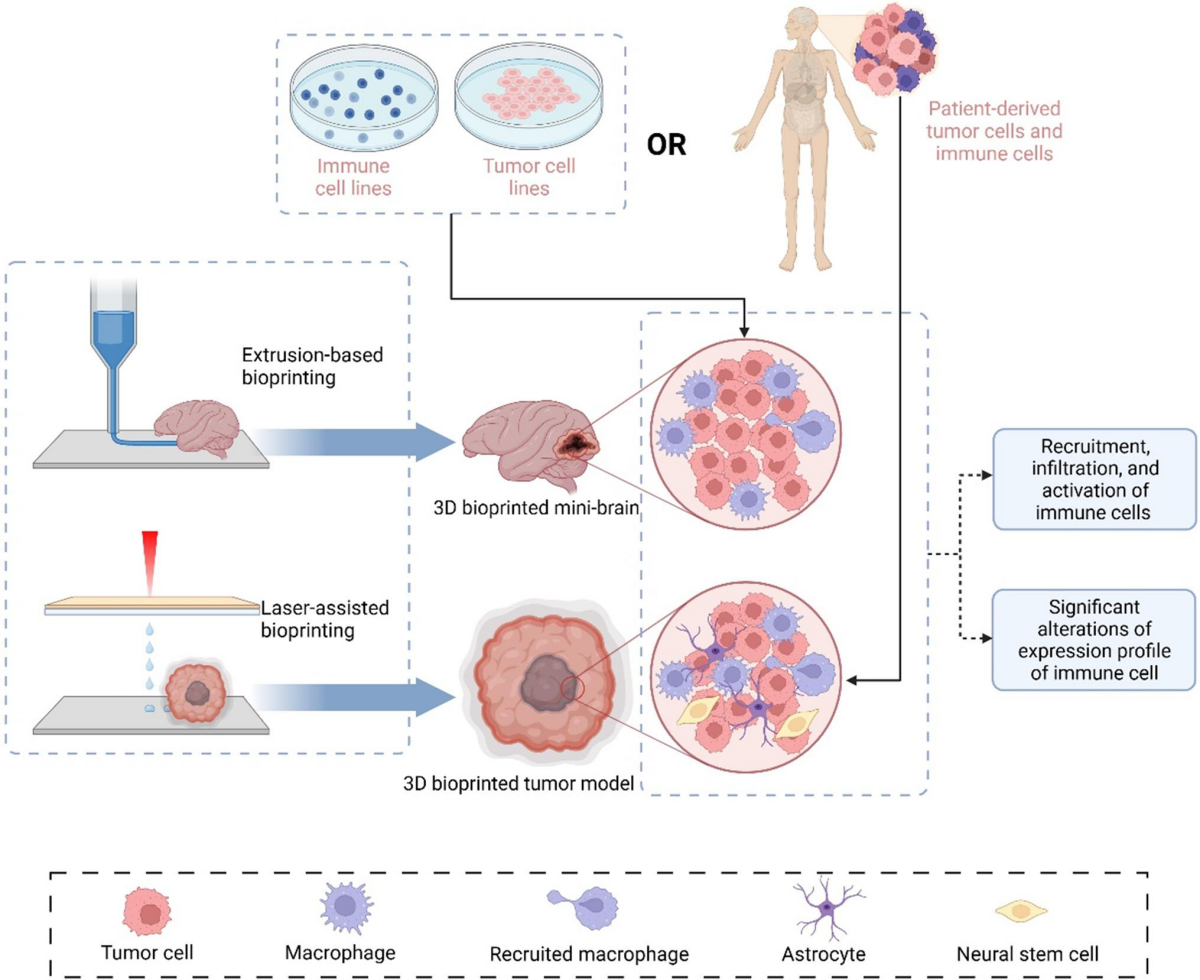
3D bioprinted tumor model reproduces crosstalk between tumor cells and immune cells. Created with Biorender.com

In addition to directly bioprinting immune cells into 3D models, infiltration of immune cells and cellular interactions can also be observed by co-culturing a 3D bioprinted tumor model with immune cells. Grunewald utilized light projection-based bioprinting technology to establish a neuroblastoma model with GelMA and observed infiltration of CD8^+^ L1CAM-specific CAR T cells dispersing from the top to bottom of the 3D tumor models. Moreover, the L1CAM-specific CAR T cells were highly activated by increased interferon gamma (IFNG) release and tumor cell cytotoxicity [69]. Moreover, Mazzaglia et al. improved the extrusion of a 3D bioprinter and termed it BioArm, which was simpler, more effective, and more portable [75]. With mM1 murine pancreatic ductal adenocarcinoma cells and pancreatic cancer-associated fibroblasts (PanCAFs) loading in a gelatin/alginate hydrogel, a core–shell tumoroid was bioprinted with mM1 cells as the core surrounded by PanCAFs. On day 5, the 3D bioprinted tumoroids were embedded within the collagen gel in which mixed population of splenocytes was presented. Upon recruitment by tumor cells and CAFs, various immune cells infiltrated the tumoroids, including T cells, NK, and CD11b^+^ cells, which interacted with the tumor cells and led to tumoroid reduction. However, the immune cells remained viable. Reproduction of interaction between tumor cells and immune cells in 3D bioprinted tumor models is demonstrated in Fig. 3.

### Simulation of crosstalk between tumor cells and non-immune stromal cells

A 3D bioprinted tumor model loaded with tumor and stromal cells also reproduces the processes of ECM remodeling, stromal cell differentiation, neovascular formation, and metastasis during tumor progression. Among stromal cells within the TME, adipocytes play important roles in supporting tumor cell growth and development. Vinson et al. studied the epithelial–adipose interaction by constructing a laser-based 3D bioprinted model of breast cancer-containing alginate–collagen microbeads and an alginate/gelatin hydrogel containing differentiated adipocytes [76]. Horder et al. used aggregate bioprinting to establish a biomimetic breast cancer model of MDA-MB-231 cells and adipose-derived stem cells (ASCs) loaded in HA-based hydrogels through extrusion-based bioprinting [80]. It was co-cultured for 9 days, and the expression profile of ASCs was altered, including upregulated collagen I and VI and fibronectin, indicating increased stromal stiffness and abnormal cellular behavior. To simulate bone metastasis, Zhou et al. utilized stereolithography-based bioprinting to fabricate a 3D biomimetic model with breast cancer cells and osteoblasts/bone marrow mesenchymal stem cells (MSCs), which were cultured in bone matrices constructed of GelMA and nanocrystalline hydroxyapatite (nHA) [77]. Compared with the monoculture, the proliferation of osteoblasts and MSCs was inhibited. However, breast cancer viability increased remarkably, indicating crosstalk between the breast cancer cells and osteoblasts/MSCs, promoting bone metastasis. Furthermore, Cui et al. developed a triculture metastatic model to study the interactions between cancer cells and vascularized bone tissue [70]. By stereolithography-based 3D bioprinting, the tumor–vessel–bone model was replicated with optimized bioinks containing breast cancer cells, osteoblasts, and human umbilical vein endothelial cells (HUVECs). This 3D in vitro model showed the migration and colonization of malignant cells toward bone after 14 days of culture.

**Fig. 4:**
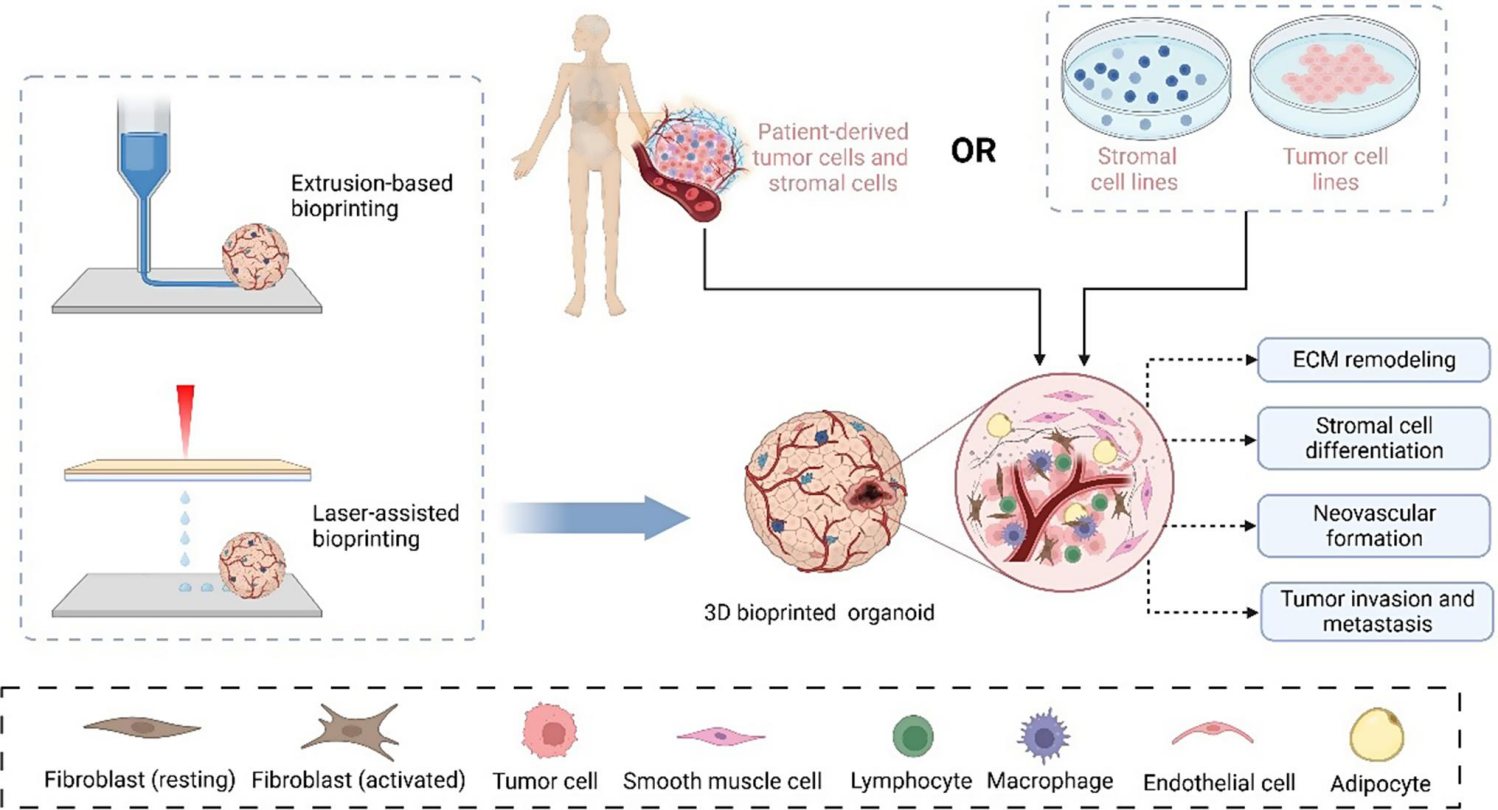
3D bioprinted tumor model reproduces crosstalk between tumor cells and non-immune stromal cells. Created with Biorender.com

Multicellular bioprinting has been widely used to establish in vitro models simulating sophisticated organ structures, such as hepatic sinus and pancreas [71, 72]. By laser-assisted bioprinting, a 3D pancreatic cell spheroid was constructed with acinar and ductal cells organized in a GelMA hydrogel, which replicated the initial stage of pancreatic ductal adenocarcinoma (PDAC) [72]. Li et al. recently built a tetra-culture model to study the interaction between cholangiocarcinoma (CCA) and stromal cells, including HUVEC, fibroblasts, and human monocyte leukemia THP-1 cells [79]. This in vitro model was constructed by extrusion-based bioprinting technology with cell-laden GelMA and recapitulated the CCA microenvironment. The interactions between cancer cells and stromal cells considerably promoted the aggressiveness of CCA. Notably, Kim et al. established a delicate 3D model to mimic the native tissue architecture and microenvironment of bladder cancer with patient-derived tumor cells and normal bladder stem cells [60]. The formed organoid was then reconstituted with four major components of bladder cancer stroma, including CAFs, epithelial cells, immune cells, and smooth muscle cells, forming an outer muscle layer. Using 3D bioprinting as a high-throughput platform, the researchers constructed a delicate aggregate-bioprinted bladder assembly that perfectly reproduced the intrinsic architectures and responses to chemotherapeutics. Moreover, tumor cells from the basal T2 stage parental tumor protruded through the stromal components and invaded the muscle layer. Recently, Neufeld et al. used fibrinogen and gelatin to develop a novel bioink composed of patient-derived glioblastoma cells, astrocytes, microglia, brain pericytes, and endothelial cells [81]. The model included bioprinted vessels that formed a perfusable vascular network, which enabled glioblastoma cells to exhibit transcriptional profiles similar to those cultured in in vivo models. Simulation of crosstalk between tumor cells and stromal cells in 3D bioprinted models is illustrated in Fig. 4.

### Simulation of interactions between tumor cells and the ECM

**Fig. 5:**
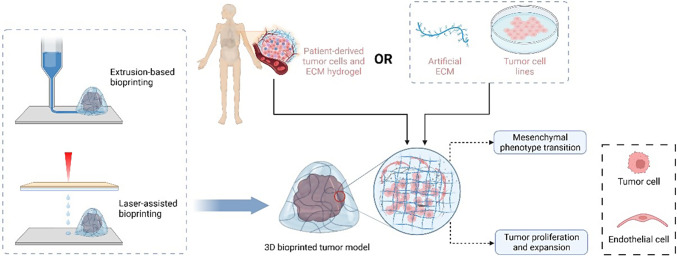
3D bioprinted tumor model recapitulates interactions between tumor cells and extracellular matrix (ECM). Created with Biorender.com

As mentioned, the biophysical features of the ECM have an important effect on tumor growth. 3D bioprinting models are designed to mimic the distinct ECM for cancer cells. Mollica et al. reported a 3D bioprinted model with a mammary-derived ECM hydrogel [82]. In this tissue-specific matrix, MCF-7 breast cancer cells and MDA-MB-231 proliferated, invaded, and formed large tumoroids after they were cultured for 14 days. Glioblastoma multiforme (GBM) is characterized by tumor heterogeneity and hypervascularization. Tang et al. recently utilized DLP-based technology to develop a 3D tri-regional GBM model with patient-derived GBM cells and human endothelial cells suspended in tissue-specific ECM-derived bioinks, which consisted of glycidyl methacrylate hyaluronic acid (GMHA) and gelatin methacrylate (GelMA) [83]. The authors observed that cellular behavior was considerably different in the TME with varied stiffness, that the soft ECM permitted rapid proliferation and expansion of GBM cells, and that the stiff ECM induced the mesenchymal phenotype transition, favoring recurrence and angiogenesis. Simulation of interactions between tumor cells and the ECM in 3D bioprinted tumor models is demonstrated in Fig. 5.

### Translational application of 3D bioprinting in tumor immunotherapy

In vitro tumor models are widely used in clinical practice to facilitate personalized medicine including tumoroid, patient-derived xenograft (PDX), and 3D bioprinting tumor model, each of which possesses its own characteristics and advantages. Tumoroid has been recognized as feasible medium-throughput drug screening platform which maintains proliferative capacity and histological features of original tumors [84]. PDX, on the contrary, is well-accepted as a qualified tumor model for drug screening which ensures complete passage of tumor cells in vivo [85]. Responses to ICIs and CAR T therapy were evaluated in PDX model of HCC, colorectal cancer, and gastric cancer which were established in humanized mice with immunodeficiency [86–88]. Moreover, glioblastoma PDX model was created by seeding patient-derived glioblastoma cells in the brain of immunodeficient mice which received transfusion of CD4+ and CD8+ T cells containing synNotch-CAR T cells after 10 days [89]. However, the establishment process of PDX model is time-consuming and high-cost rendering PDX not suitable for large-scaled clinical research [90]. Generally, PDX models in humanized mice and 3D bioprinted tumor model has their own characteristics and advantages in immunotherapy. Both models provide a microenvironment similar to the primary lesion for studying the interactions between the immune system and tumors. The PDX model is more capable of obtaining in vivo evidence and has been widely applied in recent years. However, 3D bioprinting models also have their value, as screening data can be obtained in a high-throughput manner with low economic and time costs. Therefore, 3D bioprinting tumor models and PDX models are both important and practical research strategies in tumor immunotherapy.

**Fig. 6:**
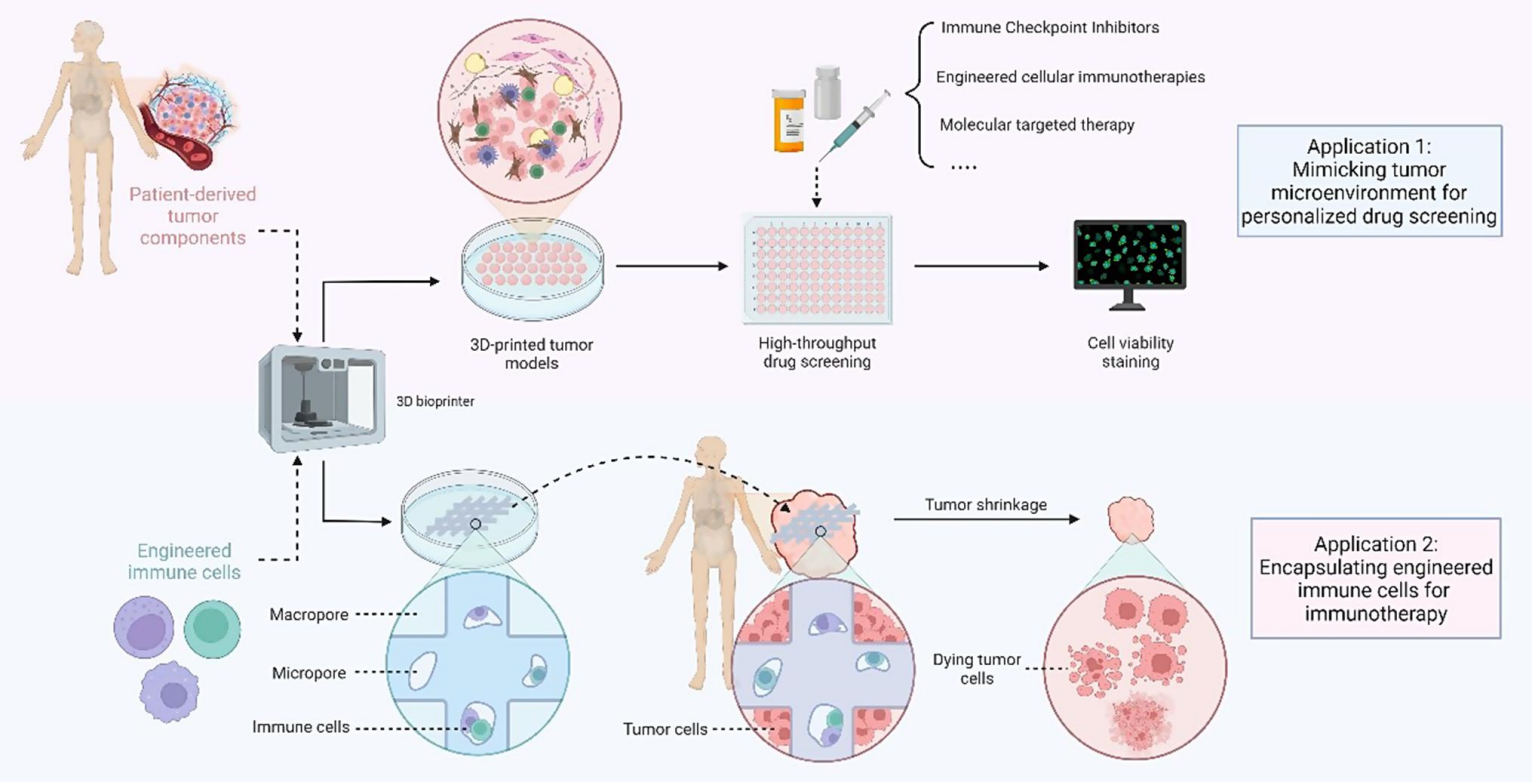
Clinical application of 3D bioprinted tumor models in immunotherapy. Created with Biorender.com

In clinical practice, 3D bioprinting has been used in combination with other in vitro tumor models to establish complex platforms with patient derived cellular components, and has been widely used for drug screening and therapeutic efficacy evaluation. Xie et al. established a 3D bioprinted model with tumor cells isolated from six HCC patients, and screened sensitive drugs for personalized treatment [17]. Yi et al. developed human-glioblastoma-on-chip with tumor cells isolated from surgery specimen, BdECM bioink, and other bioinks including vascular cell-laden BdECM bioink, to reproduce the heterogeneous ecology of glioblastoma. Subsequently, various candidate drug combinations were screened on the chip model, and effective drug combinations were reported to assist clinical design of treatment plans [91]. Similarly, 3D bioprinted bladder cancer assembloids have also been established to screen sensitive drugs in a high-throughput manner [60]. Noteworthily, a 3D bioprinting model was recently established by Kim et al., who evaluated the antitumor effects of zEGFR-CAR-NK therapy on leukemia and solid tumors. This preclinical study printed a 3D hydrogel with bioink composed of NK cells, gelatin and alginate, the structure of which consisted macropores and micropores. The macropores allowed NK cells to receive stimulus signals, and the micropores facilitated gathering of NK cells to enhance cell vitality and cytotoxic ability. When tumor cells were co cultured with hydrogel loaded with zEGFR-CAR NK cells, significant tumor killing effect was observed [92]. Clinical application of 3D bioprinted tumor models in immunotherapy is demonstrated in Fig. 6.

At present, 3D bioprinted tumor models have entered clinical research stage for purpose of treating cancer. According to data from clinicaltrials.gov, studies have been registered to investigate the application of 3D bioprinted tumor models in drug screening and personalized treatment of pancreatic ductal adenocarcinoma and colorectal cancer (NCT05955092, NCT04755907). Clinical trials using 3D bioprinted tumor models in tumor immunotherapy still need to be initiated. However, with the development and progress of materials science, it is expected that 3D bioprinting tumor model will be applied to cancer immunotherapy to screen sensitive drugs or therapies as an economic-friendly, reliable, and high-throughput platform.

## Conclusions and perspectives

Immunotherapy resistance is an unavoidable challenge in the process of tumor immunotherapy. Because tumor cells constantly evolve and change their expression profiles, a complex and dynamic network within the TME forms, including cellular crosstalk between tumor cells, immune cells, and stromal cells. The biophysical features of the ECM also contribute to tumor aggressiveness and facilitate immune evasion. Compared with the 2D culture model, 3D bioprinted tumor models have considerable advantages, and they can be utilized as optimal platforms to study cancer immunotherapy resistance. Firstly, tumor cells bioprinted in 3D models sustain in vivo aggressiveness and heterogeneity. Secondly, 3D bioprinting enables the construction of multicellular in vitro models with a tissue-specific matrix, which recapitulates the complex TME. Thirdly, 3D bioprinted tumor model can be constructed in a prompt and high-throughput manner which provides timely information for clinical reference. In these biomimetic tumor models, cell–cell and cell–matrix crosstalk can be fully demonstrated between various cellular components and the ECM. In the future, 3D bioinks contained patient-derived components are recommended to construct personalized 3D bioprinted tumor model, which will facilitate individualized medicine to conquer immunotherapy resistance. Biomimetic assembloids should also be developed through aggregate 3D bioprinting combined with other 3D culture techniques. Besides, efforts should be made to establish more sophisticated 3D bioprinted tumor models with multicellular components, in order to economically and conveniently study the interactions between tumor and TME.

## Data Availability

Not applicable.

## List of abbreviations

TME: Tumor microenvironment
ECM: Extracellular matrix
ICIs: Immune checkpoint inhibitors
ACT: Adoptive cell transfer
TILs: Tumor infiltrating lymphocytes
Tregs: T regulatory cells
TAMs: Tumor associated macrophages
MDSCs: Myeloid-derived suppressor cells
CAFs: Cancer-associated fibroblasts
TECs: Tumor endothelial cells
IFP: Interstitial fluid pressure
NSCLC: Non-small cell lung cancer
TEXs: Tumor-derived exosomes
DC: Dendritic cell
Bregs: B regulatory cells
EMT: Epithelial-mesenchymal transition
EBB: Extrusion-based bioprinting
DBB: Droplet-based bioprinting
LAB: Laser-assisted bioprinting
DLP: Digital light processing
ABB: Aspiration-based bioprinting
CLL: Chronic lymphocytic leukemia
HCC: Hepatocellular carcinoma
EGFR: Epidermal growth factor receptor
ASCs: Adipose-derived stem cells
MSCs: Mesenchymal stem cells
PDAC: Pancreatic ductal adenocarcinoma
CCA: Cholangiocarcinoma
GMHA: Glycidyl methacrylate hyaluronic acid
